# The Central Variant of Posterior Reversible Encephalopathy Syndrome: A Systematic Review and Meta-Analysis

**DOI:** 10.3390/neurolint17070113

**Published:** 2025-07-21

**Authors:** Bahadar S. Srichawla, Maria A. Garcia-Dominguez, Brian Silver

**Affiliations:** Department of Neurology, University of Massachusetts Chan Medical School, 55 Lake Ave N, Worcester, MA 01655, USA; maria.garcia-dominguez@umassmemorial.org (M.A.G.-D.); brian.silver@umassmed.edu (B.S.)

**Keywords:** PRES, central variant, posterior reversible encephalopathy syndrome, systematic review, meta-analysis, posterior reversible leukoencephalopathy syndrome

## Abstract

Background: The central variant of posterior reversible encephalopathy syndrome (cvPRES) is an atypical subtype of PRES. Although no unifying definitions exists, it is most often characterized by vasogenic edema involving “central” structures, such as the brainstem, subcortical nuclei, and spinal cord, with relative sparing of the parieto-occipital lobes. Methods: This systematic review and meta-analysis followed the PRISMA guidelines and was pre-registered on PROSPERO [CRD42023483806]. Both the Joanna Briggs Institute and New-Castle Ottawa scale were used for case reports and cohort studies, respectively. The meta-analysis was completed using R-Studio and its associated “metafor” package. Results: A comprehensive search in four databases yielded 70 case reports/series (*n* = 100) and 12 cohort studies. The meta-analysis revealed a pooled incidence rate of 13% (95% CI: 9–18%) for cvPRES amongst included cohort studies on PRES. Significant heterogeneity was observed (I^2^ = 71% and a *τ* ^2^ = 0.2046). The average age of affected individuals was 40.9 years, with a slightly higher prevalence in males (54%). The most common etiological factor was hypertension (72%). Fifty percent had an SBP >200 mmHg at presentation and a mean arterial pressure (MAP) of 217.6 ± 40.82. Imaging revealed an increased T_2_ signal involving the brain stem (88%), most often in the pons (62/88; 70.45%), and 18/100 (18%) cases of PRES with spinal cord involvement (PRES-SCI). Management primarily involved blood pressure reduction, with adjunctive therapies for underlying causes such as anti-seizure medications or hemodialysis. The MAP between isolated PRES-SCI and cvPRES without spinal cord involvement did not show significant differences (*p* = 0.5205). Favorable outcomes were observed in most cases, with a mortality rate of only 2%. Conclusions: cvPRES is most often associated with higher blood pressure compared to prior studies with typical PRES. The pons is most often involved. Despite the severity of blood pressure and critical brain stem involvement, those with cvPRES have favorable functional outcomes and a lower mortality rate than typical PRES, likely attributable to reversible vasogenic edema without significant neuronal dysfunction.

## 1. Introduction

Posterior reversible encephalopathy syndrome (PRES) is a neurological disorder characterized by a variety of symptoms, including headaches, altered mental status, seizures, and visual disturbances. It is most identified through characteristic radiological findings, predominantly involving the posterior regions of the cerebral cortex and subcortical white matter [1]. Although the exact pathophysiology of PRES remains incompletely understood, it is thought to be related to endothelial dysfunction, leading to vasogenic edema. In recent years, a variant of this condition, known as central-variant PRES (cvPRES), has been increasingly recognized. Unlike the classical form of PRES, which predominantly affects the posterior regions of the brain, central-variant PRES is characterized by the involvement of central brain structures, including the basal ganglia, thalamus, brainstem, and/or spinal cord. This variation presents unique diagnostic challenges and may have different clinical implications compared to the typical form of PRES [2]. However, no unified consensus on the definition of cvPRES has been established.

cvPRES raises several critical questions about its epidemiology, clinical presentation, risk factors, pathophysiology, and outcomes. While there is growing literature on this subject, there has been no comprehensive synthesis of the evidence to date. Misdiagnosis or delayed diagnosis can lead to inappropriate management, potentially resulting in adverse outcomes. Understanding the distinct features of central-variant PRES, including its triggers, clinical course, and response to treatment, is crucial for optimizing patient care. Additionally, the location of vasogenic edema within the posterior fossa and brainstem brings forth the concern for life-threatening herniation syndromes, which may require decompressive craniectomy and other neurosurgical interventions.

In this systematic review and meta-analysis, we aim to provide a detailed analysis of the characteristics of cvPRES, comparing them with the classical form of the syndrome where relevant. We will evaluate aspects such as demographics, clinical manifestations, precipitating factors, imaging findings, medical and surgical management strategies, purported pathophysiological mechanisms, and outcomes. By doing so, we seek to offer a comprehensive overview of this condition, contributing to better recognition, diagnosis, and management of patients with central-variant PRES.

## 2. Methods

This study was exempt from an institutional review board, as it involves analysis of de-identified data that have already been collected. Therefore, it will not be possible to trace the data presented back to individual patients.

### 2.1. Search Strategy

A PRISMA directed systematic review and meta-analysis were planned and prospectively registered on PROSPERO [CRD42023483806] and Open Science Framework (OSF) [3]. A comprehensive literature search was conducted across four electronic databases, including PubMed/PubMed Central/MEDLINE, ScienceDirect, Scopus, and Hinari. The search strategy was designed to include a combination of keywords and MeSH terms related to “Posterior Reversible Encephalopathy Syndrome,” “PRES,” “central variant,” “brainstem variant,” and related terminologies. The complete search string and terminologies for each database are included in Table 1. The search was restricted to studies published in English from the inception of the database. Reference lists of identified articles were manually searched to ensure the inclusion of additional relevant studies. Both backwards and forward citation tracking was utilized, and a gray literature search was completed by reviewing the first 100 results on Google Scholar and Open Gray.

### 2.2. Eligibility Criteria

Studies were included if they met the following criteria: (1) patients diagnosed with central-variant PRES, as defined by imaging criteria, which include increased T_2_-weighted signal involving central structures (e.g., basal ganglia, thalamus, brainstem, cerebellum, and spinal cord) in the absence of significant cortical involvement; (2) articles reporting on clinical features, imaging findings, management, and outcomes; and (3) study designs including randomized controlled trials, cohort studies, case-control studies, and case reports/series. All cases were reviewed by board-eligible or certified adult neurologists. Articles only of the English language were included. Reviews, editorials, commentaries, and studies with insufficient data on central-variant PRES were excluded. Records published in non-peer reviewed journals were not included in the analysis.

### 2.3. Data Extraction

First, the aggregate records from the initial search were exported to EndNote 21, and duplicate records were removed. Next, two reviewers (B.S.S. & M.A.G-D.) independently extracted data using the Rayyan QCRI web platform. Extracted information included study characteristics (author, year of publication, study design), patient demographics (age, gender), clinical presentations, imaging findings, management strategies, and outcomes. Discrepancies between reviewers were resolved through discussion or by consulting a third reviewer (B.S.).

### 2.4. Quality and Risk of Bias Assessment

The quality and risk of bias assessment was assessed using appropriate tools: the Joanna Briggs Institute (JBI) assessment tool for case reports/series and the Newcastle–Ottawa Scale for observational studies. The JBI tool consists of eight items addressing different aspects of the methodological quality of case reports, including precise patient demographics, accurate diagnosis, objective measurements of intervention outcomes, and follow-up information. The quality and risk of bias assessment were conducted by two authors (B.S.S., M.A.G-D.); a third author was consulted (B.S.) for any discrepancy between reviewers.

### 2.5. Statistical Analysis and Meta Analysis

Descriptive statistics were used to analyze the extracted data. Data were presented as mean ± standard deviation (SD) and as frequencies and percentages for categorical variables. In this study, we conducted a meta-analysis to estimate the incidence rate of cvPRES from cohort studies. The Shapiro–Wilk test was used to assess for normality. An unpaired t-test was used when comparing MAPs between cvPRES without spinal cord involvement (SCI) and PRES-SCI. The meta-analysis was conducted using R-Studio, employing the “metafor” package to pool the incidence rate across studies. We assessed heterogeneity among studies using the I^2^ statistic. To visualize the results, we generated forest plots to display the individual and pooled incidence rate with corresponding 95% confidence intervals. Funnel plots were used to assess publication bias, and Baujat plots were constructed to identify studies that contributed significantly to heterogeneity.

### 2.6. Data Validation

Although the data presented here and their corresponding analysis is declassified, the data sheet and document will be stored on a secure password-protected hard drive indefinitely. The ethical standards outlined by the Declaration of Helsinki will be upheld. No attempt will be made to identify individual patients.

## 3. Results

### 3.1. Search Results

**Figure 1:**
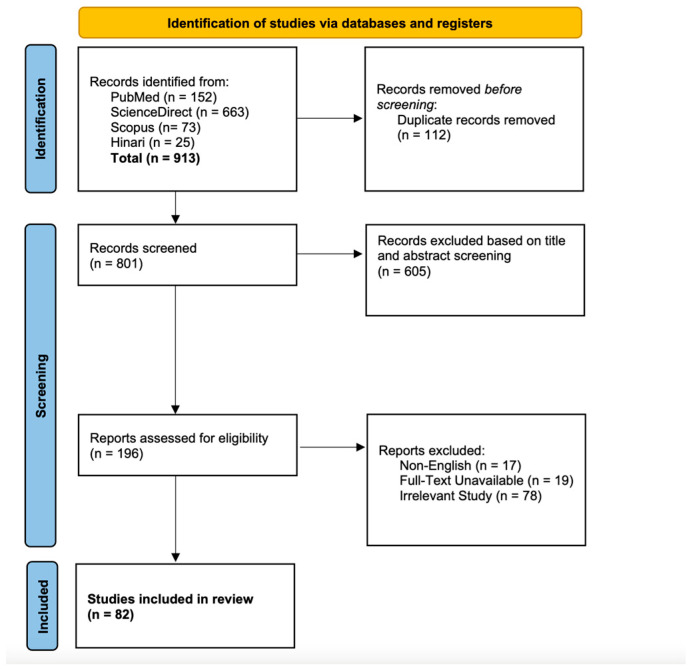
PRISMA flow diagram of included studies.

A total of 1120 records were obtained. After removing duplicates, 912 records had their abstracts and titles screened. A total of 191 records were reviewed and assessed for eligibility for inclusion in the qualitative and quantitative synthesis. A PRISMA-guided flow diagram of record assessment is provided in Figure 1. Single case records and case series are presented in Table 2 [4,5,6,7,8,9,10,11,12,13,14,15,16,17,18,19,20,21,22,23,24,25,26,27,28,29,30,31,32,33,34,35,36,37,38,39,40,41,42,43,44,45,46,47,48,49,50,51,52,53,54,55,56,57,58,59,60,61,62,63,64,65,66,67,68,69,70,71,72,73,74,75,76,77,78,79,80,81,82,83,84,85,86,87,88,89,90]. Cohort studies are included in Table 3 [7,18,21,26,62,91,92,93,94,95].

### 3.2. Clinical and Radiographic Characteristics

A total of 70 records with 100 patients are included in the single-case analysis. A total of 12 cohort studies are included. The average age of reported cases was 40.9. Fifty-four of one hundred cases occurred in men and 46/100 in women. Fourteen cases were reported in patients < 18 years of age. The most reported medical history include hypertension (40/100; 40% of cases), chronic kidney disease/end stage renal disease (ESRD) (13/100; 13%), acute kidney injury (4/100; 4%), human immunodeficiency virus (HIV) (2/100), systemic lupus erythematosus (6/100; 6%), and other immunological conditions, including Sjogren disease, polyarteritis nodosa, and thrombotic thrombocytopenic purpura (4/100; 4%). One case of T-cell acute lymphoblastic leukemia (T-ALL) (1), neurofibromatosis type-1 (NF-1), and testicular carcinoma on bleomycin, etoposide, and cisplatin were observed.

The systolic blood pressure (SBP) on arrival ranged from 120 to 270 mmHg (median: 200). Fifty out of one hundred cases had an SBP >200 mmHg. The mean SBP was 202 ± 37.58 mmHg (range: 120–270), diastolic blood pressure (DBP) 124 mmHg ± 27.35 mmHg (range: 40–220), pulse pressure (PP) 77.7 ± 22.89 (range: 36–142), and mean arterial pressure (MAP) 217.6 ± 40.82 (range: 85.3–326.66). Nineteen cases had seizures, 42/100 had headaches, and 21/100 had visual disturbance, with alteration in mental status in 14/100, three with aphasia, and one with Anton-Babinski syndrome, and 6/100 had decreased level of consciousness. Neurological symptoms localizing to the posterior circulation (e.g., dysarthria, dysphagia, dysmetria, nausea, vomiting, nystagmus, vertigo, lower cranial nerve palsy, obtundation/comatose, etc.) were seen in 30% of cases.

MRI findings included T_2_/FLAIR hyperintensities or vasogenic edema involving the brainstem in 88/100 cases: (62/88 pons, 21/88 midbrain, 22/88 medulla). Additionally, vasogenic edema was observed in other areas, including 38/100 cerebellum/cerebellar peduncle, 21/100 basal ganglia, 15/100 thalamus, and 18/100 cases involving the spinal cord (18/18 cervical cord lesion, 5/18 thoracic cord lesion). Eighteen percent of cases reported diffusion weighted imaging (DWI) hyperintensities with corresponding apparent diffusion coefficient (ADC) mapping hypointensities. Four reported susceptibility weighted imaging (SWI) hypointensities involving the bilateral thalami (1), basal ganglia (2) and pons (1), and cerebral white matter (1). These SWI findings are suggestive of chronic microbleeds due to hypertension. Shimizu et al. reported a case of pediatric T-ALL with significant vasogenic edema, diffusion restriction, and contrast enhancement within the cerebellum [68]. Yamagami et al. reported a case of central-variant PRES with associated hemorrhagic transformation involving the basal ganglia and thalamus [78]. Zhang et al. presented a case of brainstem PRES involving the pons with hemorrhagic conversion [80]. de Havenon et al. presented two cases of spinal cord PRES, which showed a central confluent lesion involving the entire spinal cord [81]. Agarwal et al. reported a pediatric case of PRES-SCI with leptomeningeal enhancement [88].

Cerebrospinal fluid analysis when completed was often normal; six cases reported elevated protein. Kachi et al. reported a case of central PRES secondary to Sjogren disease, which showed elevated SSA/B antibodies as well as elevated oligoclonal bands in CSF [41]. Decker et al. completed a cerebellar biopsy on a patient with hypertension-related central PRES and found fibrinoid change and perivascular infiltrates of mature lymphocytes [27]. The case by Yis et al. demonstrated an elevated IgG index of 0.9 (normal: <0.7) [96].

Medical management most often included blood pressure reduction and seizure management. Thirteen cases required renal-replacement therapy (RRT), one case required VA-ECMO after a cardiac arrest, one individual required plasma exchange for thrombotic thrombocytopenic purpura, and four patients were treated with intravenous methylprednisolone, often with an underlying autoimmune condition (i.e., SLE and Sjogren disease). Three cases required emergent external ventricular drain (EVD) placement, two individuals required suboccipital decompressive craniectomy, and there was one case of high cervical (C_1_) laminectomy. One patient was treated with erythropoietin, ferrous sulfate, and bicarbonate for metabolic acidosis from obstructive nephropathy leading to uremia [40]. The patient described by Vaysman et al. who was hypertensive and had SLE was treated with plasmapheresis [75]. The case by Shimizu et al. showed significant hypertension in a 10-year-old girl (>99th percentile) on a chemotherapy regimen for T-ALL including four doses of intravenous vincristine (1.5 mg/m^2^ ) and daunorubicin (30 mg/m^2^) given every week, eight doses of intramuscular L-asparaginase (5000 units/m^2^) administered every third day, daily oral dexamethasone (10 mg/m^2^/day for three weeks and subsequent taper), and a single dose of intrathecal cytarabine (30 mg), methotrexate (12 mg), and prednisolone (10 mg) administered on day five of treatment [68]. Yokoyama et al. reported a case of central-variant PRES secondary to blood pressure fluctuations from Guillain Barre syndrome (GBS) treated with intravenous immunoglobulin (IVIG) [79].

The most common purported causes were hypertension (72/100; 72%), eclampsia (2), renal failure/kidney disease (4), SLE (4), Sjogren disease (1) hypomagnesemia (1), immunotherapy (1), sulfasalazine (1), and GBS (1). The case by Chaudhari et al. reported central PRES in a one-week postpartum woman with no clear etiology [23]. The case by Sharma et al. reported a 7-year-old boy with grade IV vesicoureteral reflex with hypertension [67]. Chan et al. reported the case of a 4-year-old boy with hypertension from NF-1-related renal artery stenosis with prominent vasogenic edema involving the pons, medulla, cerebellar hemispheres, and complete spinal cord (cervical > thoracic) [87]. Marrone et al. reported a case of central-variant PRES due to hypertension secondary to renal-artery stenosis from paraaortic lymph node dissection [86]. The pediatric PRES-SCI case by Agarwal et al. occurred because of hypertension secondary to renal artery stenosis [88]. The PRES-SCI case presented by Choh et al. occurred due to hypertension from IgA nephropathy [89]. Srichawla et al. reported a case of cvPRES due to rapidly fluctuating blood pressure presumedly from adrenal insufficiency [95]. Most cases showed clinical and radiographic improvement in 1–2 months from the initial diagnosis. We report a mortality rate of 2%; all cases succumbed to the illness and died within the same hospitalization.

### 3.3. Retrospective Studies

Ahn et al. (2004) completed a single-center retrospective analysis of PRES and reported a prevalence of 4/37, 10.8% of the central-variant of PRES. Bansal et al. (2020) described 22 cases of PRES, wherein 40% had radiographic involvement of the cerebellum, 15% of the basal ganglia, 10% of the deep white matter, and 10% of the brainstem [7,18]. Brewer et al. (2013) performed a 10-year retrospective analysis on the neuroimaging findings of 47 women who had eclampsia. Eleven individuals (23%) were reported to involve the basal ganglia and cerebellum, five of whom were antepartum, and six were postpartum [97]. Chen et al. (2017) performed a retrospective analysis and found 11 individuals with the central variant of PRES (seven male and four female), with a median age of 60.0 (range 40.0–63.5). The reported etiology was hypertension in all eleven cases [91]. Chou et al. (2004) performed a retrospective analysis of 12 patients with clinical radiographic findings consistent with PRES [26]. Of the reported cases, 1/12 had radiographic findings consistent with the central variant (case 25). Fitzgerald et al. (2015) completed a retrospective review from 2007–2012 and identified 6/80 (7.5%) cases of central-variant PRES [92]. They determined a higher prevalence of extreme hypertension and renal dysfunction compared to the non-central variant [92]. Li et al. (2012) completed a retrospective study of 59 cases of PRES and reported the central variant occurring in five patients (brainstem n = 2; basal ganglia n = 3) [98]. McKinney et al. (2013) completed a retrospective review of 124 cases of PRES and determined 5/124 (4%) to have the central variant [2]. McKinney et al. (2007) completed another retrospective study of 76 PRES cases and determined that 30.3% involved the thalamus and 34.2% the cerebellum; 18.4% were within the brainstem, and 11.8% involved the basal ganglia [93]. Raman et al. (2017) completed a retrospective review of 92 patients with PRES and identified a total of 22 individuals with predominant brainstem and cerebellar involvement [99]. Yoon et al. completed a retrospective analysis of 16 PRES patients and found atypical radiographic findings including four episodes within the basal ganglia, three in the brainstem, two in the cerebellum, and three in the thalamus [100]. Aygunes et al. completed a retrospective review of 101 pediatric patients with PRES who underwent hematopoietic stem cell transplant [94]. They found a 15.8% (16/101) incidence of the central variant. They also noted a higher mortality rate (n = 10, 62.5%) compared to those with the typical variant (n = 5, 5.9%) [94].

### 3.4. PRES-SCI

A direct unpaired two-tailed *t*-test was performed to compare the mean arterial pressure (MAP) between isolated PRES with spinal cord involvement (PRES-SCI) and central-variant PRES without spinal cord involvement. Prior to the *t*-test, normality of the data was confirmed using the Shapiro–Wilk test, yielding W = 0.9329 and *p* = 0.2433, which passed the normality threshold with an α = 0.05. The unpaired t-test revealed no statistically significant difference in MAP between the two groups (t *=* 0.6453, *p* = 0.5205), suggesting that MAP levels are comparable in these presentations of PRES with spinal cord involvement. However, this may be limited by the smaller sample size in the PRES-SCI group (18 vs. 85).

### 3.5. Meta-Analysis of Incidence Rate

**Figure 2:**
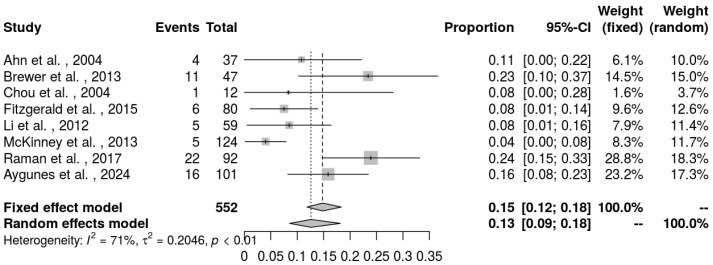
Forest plot depicting the pooled incidence rate of events across studies using both fixed-effects and random-effects models. Individual study proportions with 95% confidence intervals are shown alongside their respective weights under both models. The random-effects pooled estimate is 0.13 (95% CI: 0.09–0.18), indicating moderate heterogeneity (I^2^ = 71%, τ^2^ = 0.2046, *p* < 0.01). The diamond represents the overall effect size and its confidence interval for the random-effects model [2,7,26,92,94,97,98,99].

**Figure 3:**
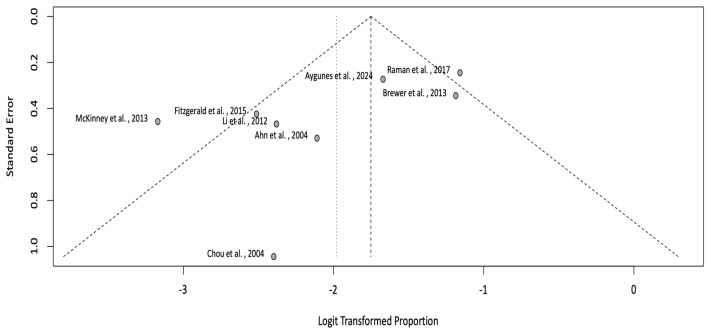
Funnel plot assessing publication bias in the included studies. The *x*-axis represents the logit-transformed proportion of events, while the *y*-axis represents the standard error. Open circles indicate individual studies, with the dashed diagonal lines representing the 95% confidence interval around the pooled effect estimate. Symmetry in the plot suggests minimal publication bias; however, asymmetry could indicate potential bias or heterogeneity in the included studies. Filled circles represent imputed studies added using the trim-and-fill method to adjust for potential bias [2,7,26,92,94,97,98,99].

**Figure 4:**
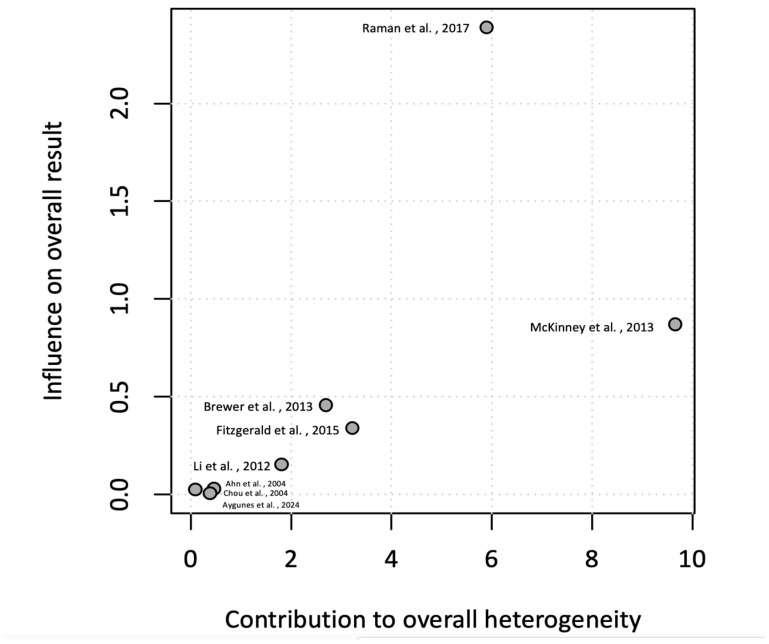
Baujat plot illustrating the contribution of individual studies to overall heterogeneity and their influence on the pooled effect size. The *x*-axis represents the contribution of each study to overall heterogeneity, while the *y*-axis indicates the study’s influence on the overall result. Studies located further from the origin exhibit a higher impact on heterogeneity and/or the pooled effect. In this analysis, McKinney et al. (2013) and Raman et al. (2017) demonstrate notable contributions to heterogeneity and influence on the pooled estimate, suggesting their potential role in driving variability within the meta-analysis [2,7,26,92,94,97,98,99].

The meta-analysis calculated the pooled incidence rate of the observed events (cvPRES) across the included studies. A total of eight studies that provided enough information on the incidence of the central variant of PRES was included in the quantitative synthesis. Using a random-effects model, the pooled incidence rate was estimated at 13% (CI: 9–18%) (Figure 2). Heterogeneity among studies was substantial, with an I^2^ = 71% and a τ^2^ = 0.2046. The heterogeneity was statistically significant (*p* < 0.01), indicating considerable variation in effect sizes across studies. To assess potential publication bias, a funnel plot was generated, and a Baujat plot was used to identify influential studies contributing to heterogeneity (Figure 3 and Figure 4).

### 3.6. Quality and Risk of Bias Assessment of Case Reports/Series

The methodological quality and risk of bias for the included studies were assessed using the Joanna Briggs Institute (JBI) critical appraisal tool. Of the studies evaluated, the majority (n = 79) achieved a perfect score (8/8), indicating high methodological rigor and a low risk of bias. These studies consistently satisfied all eight appraisal criteria, demonstrating robustness in study design and reporting. Three studies received scores of 6/8 or 5/8, reflecting a moderate risk of bias due to unmet criteria in areas such as follow-up completeness or consideration of confounding factors. These studies include Arai et al. (1997), Braatz et al. (2014), and Matsumoto et al. (2014) [10,21,51]. Two studies (Doi et al., Resorlu et al.) scored 4/8, corresponding to a high risk of bias and potential limitations in methodological quality (Table 3) [63,101]. Overall, the quality assessment demonstrates that the included studies are predominantly of high methodological quality, with a small subset requiring caution in interpretation due to moderate to high risks of bias.

### 3.7. Quality and Risk of Bias Assessment of Cohort Studies

The quality of included cohort studies was assessed using the Newcastle–Ottawa Scale (NOS), which evaluates selection (maximum 4 points), comparability (maximum 2 points), and outcomes (maximum 3 points). A total score of 9 indicates the highest quality. Many of the studies (n = 10) achieved scores of 8 or 9, indicating robust methodological quality. Notably, Chen et al. (2017) scored the maximum 9 points, meeting all criteria across selection, comparability, and outcomes [91]. Other studies, including Ahn et al. (2004), Brewer et al., and Chou et al. (2004), consistently scored 8, reflecting strong performance in most domains [7,26,97]. One study, Bansal et al. (2020), scored 4 points [18]. While it demonstrated adequacy in selection (3 points) and outcome (1 point), it lacked comparability between cohorts, which significantly impacted its total score (Table 4).

## 4. Discussion

The findings of this systematic review and meta-analysis provide a comprehensive understanding of the clinical, radiographic, and management characteristics of cvPRES, a rare but significant subtype of PRES. With an overall pooled incidence rate of 13% (95% CI: 9–18%) across cohort studies, our results underscore the clinical importance of recognizing this variant in settings where its presentation may deviate from the typical posterior-predominant PRES. In a study by Fugate et al., the mean peak systolic blood pressure among PRES patients was 199 mm Hg (range: 160–268 mm Hg), and the mean peak diastolic blood pressure was 109 mm Hg (range: 60–144 mm Hg) [102]. Another study by Rabinstein et al. reported that approximately 75% of PRES patients presented with hypertension, indicating that while elevated blood pressure is common, it is not a universal finding in PRES cases [103]. They reported a mean SBP of 182 ± 20 mm Hg and MAP 124 ± 15 [103]. In our cohort of 100 patients with cvPRES, we reported a higher mean SBP and MAP at 202 ± 37.58 mmHg and 217.6 ± 40.82, respectively.

The higher blood pressure observed in cvPRES compared to typical PRES may suggest a distinct pathophysiological mechanism potentially driven by differential vascular autoregulatory thresholds in central brain structures. Unlike the posterior cortex, which has relatively low autoregulatory capacity and is prone to vasogenic edema at moderate elevations in blood pressure, the brainstem, basal ganglia, and thalamus may require more extreme hypertension to surpass their autoregulatory limits [104]. This could explain the selective involvement of these regions in central-variant PRES. Furthermore, the proximity of these structures to deep perforating arteries with limited collateral flow may exacerbate vulnerability under conditions of severe hypertension, leading to more profound endothelial dysfunction, breakdown of the blood-brain barrier, and vasogenic edema [105]. Excessive adrenergic stimulation may amplify vascular permeability and inflammatory responses in central brain regions. Additionally, the co-occurrence of microvascular compromise or pre-existing structural abnormalities, such as chronic hypertension-induced arteriolosclerosis, could heighten susceptibility to central-variant manifestations. However, these hypotheses need to be supported by original studies.

In our dataset, the pons was predominantly affected, which may be attributed to several pathophysiological mechanisms underlying reversible pontine edema. The pons, a critical structure within the brainstem, is particularly susceptible to fluctuations in blood pressure due to its unique vascular supply and limited sympathetic innervation [106]. Acute hypertension can overwhelm the autoregulatory capacity of the pontine vasculature, leading to endothelial dysfunction and increased permeability of the blood-brain barrier [107]. Furthermore, the anatomical configuration of the pons, with its dense arrangement of neural tracts and nuclei, may predispose it to the accumulation of interstitial fluid during episodes of increased vascular permeability [52]. The resultant edema can disrupt neural conduction, leading to the clinical manifestations observed in patients with pontine involvement. Most notably, patients with cvPRES rarely reported diffusion restriction or cytotoxic edema within the pons. Thus, the impaired autoregulation does not lead to irreversible neuronal injury and permanent neurological deficits, which may be seen in a pontine stroke or central pontine myelinolysis [108].

**Table 2:** Single-patient records included in the systematic review and meta-analysis.

Case No.	Author	Publication Type	Year	Age	Gender	Risk Factors	Blood Pressure	Clinical Sequelae	CT Findings	MRI Findings	Vessel Imaging	CSF Examination	Treatment	Purported Cause	Outcomes
1	Abe et al. [4]	Case Report	2014	59	M	HTN, ESRD	181/81 mmHg	Headache, nausea, dysarthria, tic, and weakness involving the bilateral arms and legs	Hypodense lesion involving pons and cerebellum	T2/FLAIR hyperintense lesion involving the pons and cerebellum	NA	Normal	Blood Pressure management and RRT (HD)	HTN	Radiographic and clinical improvement in 13 days
2	Abraham et al. [5]	Case Report	2020	25	F	SLE, Lupus nephritis (grade IV)	SBP 190 mmHg	GTC seizure, direction-changing torsional nystagmus, horizontal ophthalmoplegia, and symmetric weakness		T2/FLAIR hyperintense and ADC hypointense lesion involving bilateral paramedian thalamic and pontine hyperintensity. SWI punctate hypointensity within the areas of restricted diffusion in the bilateral thalami and pons, compatible with microhemorrhages	MRA: Subtle short segment stenoses involving bilateral external carotid arteries, the right V4 segment, and the right P2 segment.	Normal	Blood pressure management and RRT	HTN	Clinical improvement at 3 months. SWI imaging showed persistent punctate microhemorrhages in the bilateral thalami and pons.
3	Abusabha et al. [6]	Case Report	2017	52	M	HTN	SBP 260 mmHg	Headaches, vertigo, blurred vision	NA	T2/FLAIR and DWI hyperintensities involving the bilateral cerebellum, pons, and occipital lobe	NA	NA	EVD insertion and suboccipital craniectomy and C1	HTN	Radiographic and clinical improvement in 3 weeks
4	Ahn et al. [7]	Retrospective Study	2004	29	F	NA	155/100 mmHg	Generalized tonic clonic seizures	NA	T2/FLAIR hyperintensities and ADC hypointensities involving bilateral basal ganglia	NA	NA	Blood pressure management	Postpartum eclampsia	Radiographic and clinical resolution
5	Ahn et al. [7]	Retrospective Study	2004	28	F	NA	180/120 mmHg	Generalized tonic clonic seizures	NA	T2/FLAIR hyperintensities and ADC hypointensities involving bilateral basal ganglia and thalami	NA	NA	Blood pressure management	Eclampsia	Radiographic and clinical resolution
6	Ahn et al. [7]	Retrospective Study	2004	41	M	HTN, CKD (diabetic nephropathy)	240/140 mmHg	Alteration in mental status	NA	T2/FLAIR hyperintensities and ADC hypointensities involving bilateral pons	NA	NA	Blood pressure management	HTN	Radiographic and clinical resolution
7	Ahn et al. [7]	Retrospective Study	2004	32	F	HTN, CKD	190/100 mmHg	Alteration in mental status	NA	T2/FLAIR hyperintensities and ADC hypointensities involving bilateral pons	NA	NA	Blood pressure management	HTN	Radiographic and clinical resolution
8	Akhondian et al. [8]	Case Report	2022	4	F	Secondary hyperaldosteronism, HTN.	180/110 mmHg	Alteration in mental status and status epilepticus	NA	T2/FLAIR and DWI hyperintensities in splenium of the corpus callosum, in cerebellum, brainstem, and cervical spinal cord	NA	NA	Seizure management (phenytoin, and phenobarbital). Blood pressure management (hydralazine, furosemide, and captopril).	HTN from secondary hyperaldosteronism	Radiographic and clinical resolution within one month
9	Andour et al. [9]	Case Series	2023	59	F	Diabetes, HTN	170/100 mmHg	Alteration in mental status	Normal	T2/FLAIR, DWI hyperintense and ADC hypointense lesions involving temporal and occipital regions, in corpus callosum, in the brainstem and cerebellum	NA	NA	Blood pressure management	HTN	Clinical resolution of symptoms
10	Andour et al. [9]	Case Series	2023	34	M	Kidney failure	NA	GTC seizure	NA	T2/FLAIR hyperintensity left putamen with gadolinium enhancement	NA	NA	HD	Renal failure	Clinical and radiographic resolution of symptoms
11	Arai et al. [10]	Case Report	1997	57	M	CKD, polyarteritis nodosa	200/140 mmHg	GTC seizures	Parietal lobe hypodensity	T2/FLAIR hyperintensities emporo-occipital white matter, the thalamus, the posterior limbs of the internal capsules, the external capsules, the midbrain, the pons, and the middle cerebellar peduncles	NA	NA	Blood pressure management	HTN	NA
12	Aridon et al. [11]	Case Report	2011	53	M	Thrombotic thrombocytopenic purpura	260/180 mmHg	Disturbances of gait, dizziness, urinary incontinence and lethargy	Enlargement of the lateral and third ventricles	T2/FLAIR hyperintensities involving white matter of cerebral and cerebellar hemispheres with the involvement of midbrain and cerebellar peduncles	NA	Elevated protein (77 mg/dL)	Blood pressure management (nitroprusside, furosemide, and clonidine) and plasma exchange	HTN	Clinical and radiographic resolution of symptoms at 3 months
13	Bag et al. [14]	Case Report	2010	23	F	SLE	Normal	Alteration in mental status. Being treated for SLE with IV methylprednisolone 40 mg every 8 h.		T2/FLAIR and DWI hyperintensities involving bilateral thalamic and cerebral peduncle	NA	NA	Methylprednisolone 500 mg daily and cyclophosphamide.	SLE	Clinical and radiographic resolution of symptoms at 8 months
14	Bălaşa et al. [15]	Case Report	2015	42	M	None	190/110 mmHg	Occipital headache, nausea, vomiting and disequilibrium. Elevated creatinine 9.88 mg/dL on arrival.	Multiple white matter hypodensities in both cerebellar hemispheres and the brainstem	T2/FLAIR hyperintensities involving the cerebellar white matter as well as in the pons and midbrain	NA	NA	Hemodialysis and blood pressure management	HTN (due to kidney disease)	Clinical and radiographic resolution of symptoms at 2 weeks
15	Ball et al. [16]	Case Report	2023	53	M	Alcohol abuse	NA	3 days of slurred speech, headache, and dizziness		T2/FLAIR hyperintensities involving the bilateral cerebellum	Normal	NA	Magnesium infusion	Hypomagnesemia (<0.5 mg/dL)	Clinical and radiographic resolution of symptoms (unspecified follow-up time)
16	Bandeo et al. [17]	Case Report	2018	26	F	Ulcerative colitis Adalimumab (40 mg every other week)	NA	Thunderclap headache with photophobia, nausea, and vomiting.	NA	Left frontal cSAH and hyperintense lesions on T2-weighted and FLAIR sequences located in both occipital lobes, left cerebellar hemisphere, and brainstem	DSA: Normal	NA	Discontinuation of adalimumab	Adalimumab	Clinical and radiographic resolution of symptoms at 2 months
17	Bansal et al. [18]	Retrospective Study	2020	16	F	Viral hepatitis, and acute kidney injury	NA	Alteration in mental status, seizures, vomiting	NA	T2/FLAIR hyperintensities including basal ganglia, deep white mater and temporal lobe	NA	NA	Blood pressure management	HTN	NA
18	Barnaure et al. [19]	Case Report	2014	57	M	HTN	Elevated (unspecified)	Gait ataxia	NA	T2/FLAIR hyperintensities involving e pons, medulla, and cerebellum, and a small zone of contrast enhancement in the pons	NA	Normal	Blood pressure management	HTN	Clinical and radiographic resolution of symptoms at 1 week
19	Bing et al. [20]	Case Report	2019	53	M	None	220/120 mmHg	Acute onset aphasia, fever	NA	T2/FLAIR hyperintensities involving the pons, medulla, and cervical spinal cord	NA	NA	Blood pressure management (nicardipine and urapidil)	HTN	Clinical and radiographic resolution of symptoms in one month
20	Braatz et al. [21]	Case Report	2014	39	M	NA	230/140 mmHg	Headache, nausea, blurriness, and cortical blindness	NA	T2/FLAIR and DWI hyperintensities involving the pons and medulla	NA	NA	Blood pressure management	HTN	NA
21	Chakroun-Walha et al. [22]	Case Report	2016	14	M	Renal insufficiency	200/150 mmHg	Headaches, ataxia, hemianopsia, bilateral strabismus, GTC seizure	Hypodensities in the brainstem	T2/FLAIR hyperintensities involving the brainstem and partially cerebellum	NA	NA	Blood pressure management and hemodialysis	HTN (Renal etiology)	Persistent strabismus and hemianopsia
22	Chaudhari et al. [23]	Case Report	2018	28	F	Caesarean section one week prior to presentation	NA	Seizure (abnormal movements of limbs and tongue-biting)		T2/FLAIR hyperintensities involving bilateral basal ganglia, and left cerebellum	NA	Normal	Seizure management (levetiracetam, and phenytoin)	Postpartum?	Clinical and radiographic improvement in 8 weeks
23	Chen et al. [109]	Case Report	2014	55	M	HTN	210/140 mmHg	Headache and dizziness	NA	T2/FLAIR hyperintensities in the pons, midbrain bilateral thalami, and cerebellar hemispheres with multiple microbleeds at bilateral basal ganglia	NA	NA	Blood pressure management	HTN	Clinical and radiographic improvement in 1 month
24	Chiang et al. [25]	Case Report	2019	47	M	ESRD, missed three dialysis sessions	194/114 mmHg	Comatose	Hypodentisities in the brainstem	T2/FLAIR hyperintensities involving the pons and cerebellum	NA	Normal	RRT and blood pressure management	HTN (missed dialysis sessions)	Clinical and radiographic improvement in 2 weeks
25	Chou et al. [26]	Retrospective Study	2004	52	M	HTN	194/120	Seizure, aphasia	NA	T2/FLAIR hyperintensities involving deep white matter, thalamus, and pons	NA	NA	Blood pressure management, anticonvulsants, and RRT.	HTN	Clinical and radiographic improvement in 4 months
26	Decker et al. [27]	Case Report	2009	74	M	HTN	224/144 mmHg	Slurred speech, right-sided facial droop, and incontinence of urine	NA	T2/FLAIR hyperintensities involving the brainstem (pons and midbrain)	NA	Normal (opening pressure of 16 cm of water, a protein of 129 mg/dL, WBC count of 1/mm^3^ , and glucose of 65 mg/dL (serum glucose 137 mg/dL).	Blood pressure management	HTN	Clinical and radiographic improvement in 3 months
27	Deguchi et al. [28]	Case Report	2012	42	F	HTN, CKD, thrombocytopenia	270/150 mmHg	Headache, nausea	NA	T2/FLAIR hyperintensities involving the brainstem and cerebellum	NA	NA	Blood pressure management (amlodipine, nifedipine, arotinolol)	HTN	Clinical and radiographic improvement in 1 month
28	Dhawan et al. [29]	Case Report	2010	10	F	Pheochromocytoma	260/190 mmHg	Facial palsy and bilateral papilledema	NA	T2/FLAIR hyperintensities in the bilateral caudate, putamen, thalamus, left-sided posterior limb of internal capsule, midbrain	NA	NA	Blood pressure management	HTN (pheochromocytoma)	Clinical and radiographic improvement in 2 years
29	Di Stefano et al. [30]	Case Report	2019	46	M	Mononucleosis, GERD	200/140 mmHg	One-month history of headache and blurry vision in the right eye	NA	T2/FLAIR hyperintensities involving brainstem (midbrain, pons, medulla) and cervical spinal cord (C5–C6)	Normal	NA	Blood pressure management (ramipril, doxazosin)	HTN	Clinical and radiographic improvement in 3 weeks
30	Doi et al. [101]	Case Report	2020	73	M	Metastatic colorectal cancer	NA	Acute onset visual disturbance	NA	Increased T2-weighted signal within the pons, cerebellum, and bilateral optic nerves. Diffusion restriction within the pons and cerebellum	NA	NA	NA	NA	NA
31	Doi et al. [31]	Case Report	2006	35	M	None	180/118 mmHg	Headache, nausea, blurred vision	NA	Increased T2-weighted signal in the pons, cerebellum, and basal ganglia	NA	NA	Blood pressure management	HTN	Clinical and radiographic improvement in 1 month
32	Doi et al. [31]	Case Report	2006	52	F	HTN	200/130 mmHg	Altered mental status	NA	Increased T2-weighted signal in the midbrain, basal ganglia, and cerebellum	NA	NA	Blood pressure management	HTN	Radiographic improvement in 2 weeks
33	Fujii et al. [32]	Case Report	2023	57	M	None	173/134 mmHg	Rigidity	NA	Increased signal on T2WI within the bilateral cerebellum, optic tract, cerebellar vermis, and cervical spinal cord. With DWI and ADC correlate in most lesions	NA	NA	RRT and blood pressure management	HTN	Radiographic improvement in five weeks
34	Gowan et al. [33]	Case Report	2019	47	F	HTN, T2DM, ESRD	180/99 mmHg	1-week history of altered mental status, difficulties walking, fatigue, and syncope	Normal	Increased signal on T2WI involving the pons and cerebellum	NA	NA	RRT and blood pressure management	HTN	Radiographic improvement on day 9
35	Grossbach et al. [34]	Case Report	2014	65	F	Colon cancer	217/113 mmHg	Comatose		Increased signal on T2WI involving the bilateral cerebellum	NA	CSF: Normal	Mechanical ventilation, sub-occipital craniectomy, EVD placement,	HTN	Modified Rankin score (mRS) 0 in six months
36	Hama et al. [35]	Case Report	2019	49	F	CKD	242/144 mmHg	2-week history of vomiting and malaise	NA	Increased signal on T2WI involving the pons and medulla	NA	NA	RRT and blood pressure management	HTN and kidney disease	Clinical and radiographic improvement in 3 weeks
37	Han et al. [36]	Case Report	2019	46	M	HTN	147/103 mmHg	Acute onset dysarthria and mild dysphagia	NA	Increased signal on T2WI brainstem, cerebellum and corticospinal Tracts. Diffusion restriction involving pons. Microhemorrhages seen on SWI within pons	NA	CSF: Elevated protein 55 mg/dL	Conservative management	Acute kidney injury	Clinical and radiographic improvement within 4 weeks
38	Hayashi et al. [37]	Case Report	2022	71	M	NA	209/124 mmHg	Decreased level of consciousness	NA	Increased signal on T2WI within the pons	NA	NA	Blood pressure management	HTN	Clinical improvement and discharge on day 20
39	Hebant et al. [38]	Case Report	2019	80	F	HTN, HLD	190/110 mmHg	Acute alteration in mental status	NA	Increased signal on T2WI involving the medulla and right cerebellar peduncle	NA	CSF: Elevated protein.	Blood pressure management	HTN	Clinical and radiographic improvement within a few days
40	Ho et al. [12]	Case Report	2016	49	M	NA	202/138 mmHg	Vertigo, cognitive decline, and difficulty ambulating	Hypodensity involving pons	Increased signal on T2WI involving the pons	NA	NA	Blood pressure management	HTN	Radiographic resolution in 10 days, clinical improvement in
41	Honda et al. [13]	Case Report	2019	46	F	NA	208/140 mmHg	Visual impairment	NA	Increased signal on T2WI involving the pons and bilateral cerebellar hemispheres	NA	NA	Blood pressure management	HTN	Clinical and radiographic resolution
42	Jesrani et al. [39]	Case Report	2021	39	F	NA	118/74 mmHg	GTC seizures	NA	Increased signal on T2WI involving the right caudate nucleus, bilateral thalami, and left globus pallidus	NA	NA	Anti-seizure medications	SLE	Death on day 4
43	Jia et al. [40]	Case Report	2017	14	F	Neurogenic bladder	120/81 mmHg	Decreased level of consciousness (comatose)	NA	Increased signal on T2WI involving the midbrain and pons	NA	NA	Erythropoietin, ferrous sulfate, bicarbonate	Renal failure (obstructive nephropathy)	Discharged on day 14 at baseline and radiographic resolution at 2 months
44	Kachi et al. [41]	Case Report	2023	71	F	HTN, Sjogren’s disease	197/108 mmHg	Unsteadiness and weakness of the left lower extremity	NA	Increased signal on T2WI involving the basal ganglia, thalamus, brainstem, cerebellum	NA	CSF: Elevated protein, oligoclonal bands anti-SSA/SSB, IL-6	IVMP 1000 mg for 3 days. Followed by oral prednisone 1 mg/kg.	Sjogren disease	Significant improvement on discharge
45	Katano et al. [42]	Case Report	2010	54	M	NA	201/113 mmHg	Dysarthria and altered mental status	NA	Increased signal on T2WI involving the pons with ADC correlate	NA	NA	Antihypertensive therapy with hemodialysis	HTN	Continued renal dysfunction, unspecified neurological outcome
46	Kitaguchi et al. [43]	Case Series	2005	73	M	CKD, Wernicke encephalopathy	220/116 mmHg	Appetite loss and failure to thrive	NA	Increased signal on T2WI involving the pons, thalamus, and cerebellum	NA	NA	Antihypertensive therapy	HTN	Mild lateral gaze palsy at 16-month follow-up
47	Kitaguchi et al. [43]	Case Series	2005	49	F	Aortic valve replacement	88/40 mmHg	GTC seizure, flu-like symptoms	CT scan showed a lacunar infarction in the left internal capsule of the brain	Increased signal on T2WI involving the pons	NA	CSF: Normal	Supportive treatment	Undefined	Clinical and radiographic improvement in 2 months. Residual lacunar infarction within pons
48	Lamotte et al. [44]	Case Report	2021	51	M	Nasopharyngeal carcinoma	185/125 mmHg	Dysarthria and gait instability	NA	Increased signal on T2WI involving the bilateral cerebellum, pons, and temporal lobes	NA	NA	Hemodialysis	Acute renal failure	Resolution of neurological symptoms in 3 days
49	Lee et al. [45]	Case Report	2017	47	F	NA	270/220 mmHg	AMS	NA	Increased signal on T2WI involving the pons	NA	CSF: Opening pressure 21 cm H_2_O. Protein 102 mg/dL, albumin 64 mg/dL.	Blood pressure management	HTN	Resolution of neurological symptoms in 3 days and radiographic resolution on 9th day
50	Liu et al. [46]	Case Report	2018	37	F	HTN	240/140 mmHg	Headache and blurry vision	NA	Increased signal on T2WI with diffusion restriction involving the midbrain	Normal	NA	Blood pressure management	HTN	Clinical and radiographic resolution in 2 weeks
51	Maciel et al. [47]	Case Report	2015	44	F	NA	150/110 mmHg	Subacute onset headache and visual disturbance	NA	Increased signal on T2WI involving the midbrain, pons, medulla, and cerebellar hemispheres	NA	NA	Blood pressure management	HTN	Clinical resolution in 2 weeks
52	Maier et al. [49]	Case Report	2018	22	F	SLE	Normal	GTC seizures	Symmetrical hypodense lesions within the basal ganglia	Increased signal on T2WI involving the bilateral basal ganglia	NA	NA	Anti-seizure medications and osmolar therapy	SLE associated Immunosuppressive therapy (cyclophosphamide and azathioprine)	Death due to septic shock
53	Malhotra et al. [48]	Case Report	2017	42	F	SLE, HTN, pulmonary hypertension	217/75 mmHg	Confusion, dysarthria, R hemiparesis, and hemianesthesia	Normal	Increased signal on T2WI involving the bilateral basal ganglia and thalamus	DSA: Normal	EEG: Normal	Blood pressure management	HTN	Radiographic improvement at 5 weeks and discharge to a long-term care facility
54	Maruyama et al. [50]	Case Report	2023	53	M	HTN	214/145 mmHg	Headache and left-sided weakness	NA	Increased signal on T2WI involving the pons and bilateral cerebellar hemispheres	NA	NA	Blood pressure management	HTN	Radiographic improvement in 3 weeks and clinical resolution at 8 weeks
55	Matsumoto et al. [51]	Case Report	2023	70s	F	HTN	199/111 mmHg	Generalized weakness	NA	Increased signal on T2WI involving the pons, cerebellum, and medulla	NA	NA	Blood pressure management	HTN	Radiographic resolution in 10 months
56	McCarron et al. [52]	Case Report	2008	42	M	NA	195/115 mmHg	GTC seizure, right-sided hemiparesis	NA	Increased signal on T2WI involving the pons	NA	NA	Blood pressure management	HTN	Almost complete radiographic resolution at 3-month follow-up
57	Moosa et al. [53]	Case Report	2011	8	F	Cloacal exstrophy, omphalocele, Chiari malformation with myelomeningocele and syringomyelia, and renal dysplasia with end stage renal disease.	180/120 mmHg	Status epilepticus	NA	Increased signal on T2WI involving the midbrain and pons	NA	NA	Blood pressure management and anti-seizure medications	HTN	Radiographic resolution in 9 days
58	Nagato et al. [54]	Case Report	2009	14	F	NA	185/145 mmHg	Nausea, vomiting, abdominal pain	NA	Increased signal on T2WI involving the pons, medulla, cerebellum, and cervical spinal cord	NA	NA	Blood pressure management	HTN	Significant clinical and radiographic improvement over 5 months
59	Nanba et al. [55]	Case Report	2016	47	F	NA	197/106 mmHg	Headache	NA	Increased signal on T2WI involving the pons, bilateral thalamus, bilateral basal ganglia, and periventricular white matter	NA	NA	Blood pressure management	HTN	Radiographic improvement in 2 weeks
60	Navarro-Ballester et al. [56]	Case Report	2021	62	M	HTN, HLD	190/95 mmHg	Nausea and vomiting	NA	Increased signal on T2WI involving the midbrain with SAH	DSA: mildly hypoplastic right vertebral artery	NA	Blood pressure management	HTN	Modified Rankin score of one at 6-month follow-up
61	Ocek et al. [57]	Case Report	2015	55	F	Psoriatic arthritis	120/80 mmHg	Seizure, headache, hemiparesis	NA	Increased signal on T2WI involving the basal ganglia and thalamus	NA	CSF: Normal. EEG: Mild background slowing	Removal of toxic agent	sulfasalazine	Radiographic resolution of symptoms in 1 month
62	Ogaki et al. [66]	Case Report	2009	49	F	NA	260/170 mmHg	Subacute onset worsening blurry vision	NA	Increased signal on T2WI involving the pons, bilateral cerebellum, and thalami	NA	NA	Blood pressure management	HTN	Discharge in one month with radiographic improvement
63	Ohashi et al. [58]	Case Report	2022	4-month-old	F	NA	SBP 100–130 mmHg	Cardiac arrest following immunization	NA	Increased signal on T2WI involving the bilateral basal ganglia	NA	NA	VA-ECMO, peritoneal dialysis	HTN	Discharge in 6 months
64	Onomura et al. [59]	Case Report	2022	40s	F	Hypertension	230/150 mmHg	Headache, fatigue, and nausea	NA	Increased signal on T2WI involving the supratentorial white matter, cerebellum, pons, and cerebellar peduncles. Numerous white matter microhemorrhages involving the cerebral white matter	NA	NA	Blood pressure management	HTN	Discharge on day 20 with moderate improvement
65	Osman et al. [60]	Case Report	2013	32	M	T1DM, HTN	220/140 mmHg	GTC seizure	NA	Increased signal on T2WI involving the bilateral pons and midbrain	NA	CSF: Normal	Blood pressure management	HTN	Repeat neuroimaging 12 days later showed significant improvement
66	Ou et al. [61]	Case Report	2018	40	M	NA	200/140 mmHg	Headache	NA	Increased signal on T2WI involving the pons	NA	NA	Blood pressure management	HTN	Clinical resolution within three days
67	Raya et al. [62]	Case Report	2019	29	M	HIV, ESRD, HTN	245/141 mmHg	Headache and burry vision	NA	Increased signal on T2WI involving the bilateral cerebellar hemisphere with more subtle involvement of the bilateral occipital lobes	NA	NA	Blood pressure management	HTN	Discharged on day 7 of hospitalization with no symptoms
68	Resorlu et al. [63]	Case Report	2017	39	M	HTN	170/110 mmHg	Headache	NA	Increased signal on T2WI involving the pons	NA	NA	Blood pressure management	HTN	Radiographic resolution on hospital day 20
69	Ribeiro et al. [65]	Case Report	2013	59	F	HIV	210/110 mmHg	Headache, nausea, vomiting, blurry vision	NA	Increased signal on T2WI involving the basal ganglia, thalamus, internal and external capsules, and pons. SWI: microhemorrhages within the pons	NA	NA	Blood pressure management	HTN	Clinical improvement on hospital day 3
70	Sallah et al. [64]	Case Report	2021	64	F	ESRD, stroke	207/110 mmHg	Lethargy	NA	Increased signal on T2WI involving the pons and bilateral middle cerebellar peduncles	NA	EEG: Findings within the ictal-interictal continuum.	Blood pressure management and hemodialysis	HTN + ESRD	Clinical and radiographic resolution on day 11 of hospitalization
71	Sharma et al. [67]	Case Report	2017	7	M	None	190/100 mmHg	GTC seizure	NA	Increased signal on T2WI involving the midbrain, pons, medulla, and cervical spinal cord	NA	NA	Blood pressure management and anti-seizure medications	HTN + Grade IV vesicoureteral reflex.	Radiographic improvement within 3 weeks
72	Shimizu et al. [68]	Case Report	2013	10	F	T-ALL	141/105 mmHg	Headache	NA	Increased signal on T2WI involving the bilateral cerebellum. Gd+ enhancement within the same area. DWI showing patchy diffusion restriction within the bilateral cerebellum	NA	NA	Blood pressure management	HTN+ chemotherapy (vincristine, danorubicin, dexamethasone, cytarabine, methotrexate, prednisolone)	Radiographic improvement within 6 months
73	Srinivasan et al. [69]	Case Report	2017	71	M	HTN	200/140 mmHg	Headache, dizziness, loss of consciousness	NA	Increased signal on T2WI involving the pons with restricted diffusion on DWI	NA	NA	Blood pressure management	HTN	Clinical improvement. Repeat imaging not completed.
74	Tan et al. [70]	Case Report	2019	52	M	HTN, CKD	267/159 mmHg	Headache, dizziness	CTH: Hypoattenuation involving the pons	NA	NA	NA	Blood pressure management	HTN	Clinical improvement on day 2 of hospitalization
75	Tari Capone et al. [71]	Case Report	2014	37	M	HTN	270/160 mmHg	Headache, and blurry vision	NA	Increased signal on T2WI involving the midbrain and pons	NA	NA	Blood pressure management	HTN	Clinical improvement in 2 weeks and radiographic resolution within 3 months
76	Thambisetty et al. [72]	Case Series	2003	38	M	HTN, anemia, thrombocytopenia, chronic hyponatremia, ETOH abuse	210/130 mmHg	Headache, right-sided weakness	Obstructive hydrocephalus, non-enhancing hypoattenuation in pons, midbrain	Obstructive hydrocephalus, increased T2 signal in the pons and midbrain	NA	NA	Blood pressure management	HTN	Clinical resolution
77	Thambisetty et al. [72]	Case Series	2003	61	M	None	219/138 mm.Hg	Left-sided weakness	Old lacunar infarction in anterior limb of right internal capsule, 5-mm hemorrhagic focus in right putamen	Increased T2 signal in the pons, cerebral peduncles and basal ganglia bilaterally	NA	NA	Blood pressure management	HTN	Clinical resolution
78	Thambisetty et al. [72]	Case Series	2003	46	M	T2DM, HTN, anemia, stroke	203/139 mmHg	Blurred vision, confusion	Hypoattenuation in the pons, cerebral peduncles and internal capsule bilaterally	Increased T2 signal in midbrain, pons, medulla and cerebral peduncles. Focus of hemorrhage in left basal ganglia	NA	NA	Blood pressure management	HTN	Clinical resolution
79	Tortora et al. [73]	Case Report	2015	32	F	Abortion (1-month ago)	120/70 mmHg	Headache and fever	Hypoattenuation within the right basal ganglia	Increased T2 signal and diffusion restriction in the pons	NA	NA	Enoxaparin and magnesium sulfate	Unknown	Residual coordination deficits of the extremities, and radiographic findings of pontine ischemia
80	Tsutsumi et al. [74]	Case Report	2012	54	F	None	260/142 mmHg	Dysarthria, right-sided hemiparesis	Normal	Increased T2 signal involving the pons. MR spectroscopy showed an elevated choline level	NA	NA	Blood pressure management	HTN due to pseudochromocytoma	Radiographic resolution on hospital day 18
81	Tsutsumi et al. [74]	Case Report	2012	44	F	None	209/130 mmHg	Headache, blurry vision, gait abnormalities	NA	Increased T2 signal involving the pons	NA	NA	Blood pressure management	HTN	Radiographic resolution on hospital day 20
82	Vaysman et al. [75]	Case Report	2019	22	F	SLE	197/121 mmHg	Headache, and joint pain	Normal	Increased T2 signal involving the midbrain	NA	NA	Blood pressure management and plasmapheresis	HTN + SLE	Clinical and radiographic resolution of the patients’ symptoms on day 10
83	Wakely et al. [76]	Case Report	2005	33	M	None	210/150 mmHg	Headache, and visual disturbance	Normal	Increased T2 signal and diffusion restriction involving the pons	NA	NA	Blood pressure management	HTN	Radiographic resolution of symptoms within 2 months
84	Wittgrove et al. [77]	Case Report	2018	57	M	None	220 mmHg	Aphasia, altered mental status, right-sided weakness	Normal	Increased T2 signal involving the pons and cerebellar peduncles	NA	Elevated protein 80 mg/dL	Blood pressure management	HTN secondary to renal artery stenosis	Clinical and radiographic improvement of the patient’s symptoms
85	Yamagami et al. [78]	Case Report	2019	41	F	HTN	237/142 mmHg	Headache	Hemorrhage involving the left thalamus and basal ganglia	Increased T2 signal involving the left cerebellum, pons, temporal lobes, and bilateral basal ganglia	DSA: Normal	NA	Blood pressure management	HTN	Transferred to an inpatient rehabilitation facility on hospital day 40 with a modified Rankin score of 3. Repeat MRI showing resolution of vasogenic edema
86	Yis et al. [96]	Case Report	2016	9	F	None	225/110 mmHg	Headache, vomiting, visual disturbance	NA	Increased T2 signal involving the medulla and cervical spinal cord	NA	CSF: 57 mg/dL, IgG Index 0.9.	Blood pressure management and methylprednisolone	HTN	Clinical improvement in 7 days and radiographic resolution in 10 days
87	Yokoyama et al. [79]	Case Report	2019	43	M	Guillain Barre syndrome (GBS)	152/88 mmHg	Decreased level of consciousness	NA	Increase T2 signal involving the pons, midbrain, cerebellar peduncle, and basal ganglia. Diffusion restriction	NA	NA	IVIG and blood pressure management	HTN secondary to GBS	Significant improvement with residual limb weakness
88	Zhang et al. [80]	Case Report	2016	35	M	HTN	200/140 mmHg	Dizziness	Hypodensity involving the pons with focal hyperdensity consistent with acute hemorrhagic conversion	Increased T2 signal involving the pons	NA	CSF: Elevated opening pressure of 245 mm H_2_O	Blood pressure management	HTN	Clinical and radiographic improvement in one month
89	de Havenon et al. [81]	Case Report	2014	50	M	HTN, CKD	180/110 mmHg	Headache, vomiting, confusion	NA	Increased T2 signal involving the parieto-occipital lobes, bilateral cerebellum, medulla and confluent central lesion involving the entire spinal cord	NA	NA	Blood pressure management	HTN	Near complete radiographic resolution 5 months. Clinically has residual mild lower extremity weakness
90	de Havenon et al. [81]	Case Report	2014	25	M	HTN	225/160 mmHg	Headache, vision loss	NA	Increased T2 signal involving the medulla and central lesions involving the entire spinal cord	NA	NA	Blood pressure management	HTN	Complete clinical and radiographic resolution in 3 months
91	Milia et al. [82]	Case Report	2008	44	F	HTN	240/140 mmHg	Headache, lower extremity weakness, blurry vision	NA	Increased T2 signal involving the pons, medulla, and a central cord lesion from the cervicomedullary junction to C5	NA	CSF: Normal	Blood pressure management	HTN	Clinical and radiographic resolution in 6 months
92	Samara et al. [83]	Case Report	2019	42	M	HTN	250/130 mmHg	Headache and blurry vision	Periventricular edema, enlarged ventricles, and effacement of the basilar cistern	Increased T2 signal involving the medulla, cerebellum, and upper cervical spinal cord.	NA	CSF: Normal	Blood pressure management	HTN	Clinical and radiographic resolution in 2 months
93	Liu et al. [84]	Case Report	2019	20	M	HTN	260/140 mmHg	Blurry vision and weakness	NA	Increased T2 signal involving the medulla, cervical and thoracic spinal cord	NA	NA	Blood pressure management	HTN	Clinical and radiographic resolution in 10 days
94	Chan et al. [87]	Case Report	2018	4	M	NF1	180/80 mmHg	Tachycardia	NA	Increased T2 signal involving the pons, medulla, cerebellum, and complete spinal cord (most prominent in cervical cord)	NA	CSF: Normal	Blood pressure management	HTN from NF 1-related renal artery stenosis	Clinical and radiographic improvement in 6 weeks with some residual vasogenic edema within the medulla
95	Gocmen et al. [85]	Case Report	2016	10	M	HTN, ESRD	170/120 mmHg	Headaches	NA	Increased T2 signal involving the medulla and cervical spinal cord (C_1_-C_5_)	NA	Deferred	Blood pressure management	HTN from ESRD	Clinical and radiographic resolution in 10 days
96	Marrone et al. [86]	Case Report	2016	19	M	Testicular carcinoma (bleomycin, cisplatin and etoposide)	240/140 mmHg	Headache, nausea, and central scotoma of the left eye	NA	Increased T2 signal involving the bilateral basal ganglia, pons, medulla, and anterior portion of the entire cervical spinal cord	NA	NA	Blood pressure management	HTN from renal artery stenosis from paraaortic lymph node dissection	Clinical and radiographic resolution in two weeks
97	Agarwal et al. [88]	Case Report	2016	14	M	Appendectomy	170/110 mmHg	Seizure, headache, and blurry vision	NA	Increased T2 signal involving the pons, medulla, and entire spinal cord. Contrast sequence showing diffuse leptomeningeal enhancement	NA	CSF: Normal	Blood pressure management	HTN from renal artery stenosis	Clinical and radiographic improvement in 28 days
98	Choh et al. [89]	Case Report	2011	17	M	None	240/130 mmHg	Headache, visual disturbance, vomiting	NA	Increased T2 signal involving the medulla and cervical spinal cord	NA	NA	Blood pressure management	HTN from IgA nephropathy	Clinical and radiographic improvement within one month
99	Khokhar et al. [90]	Case Report	2016	22	M	None	200/140 mmHg	Headache, vomiting, blurry vision	NA	Increased T2 signal involving the parieto-occipital lobes with predmoninant involvement of the medulla, cerebellar hemisphere, and cervical spinal cord	NA	CSF: Normal. EEG: Normal	Blood pressure management	HTN	Clinical and radiographic improvement in 25 days
100	Srichawla et al. [95]	Case Report	2025	59	F	HTN, COPD	195 mmHg SBP	Seizures, altered mental status	NA	Increased T2 signal involving the bilateral cerebellar hemisphere	CTA: Normal	CSF: Normal. EEG: Bilateral intermittent rhythmic discharges. Generalized periodic discharges, lateralized rhythmic delta activity in the left frontal lobe.	Blood pressure management	HTN due to adrenal insufficiency	Clinical and radiographic improvement on day 15

ADC: apparent diffusion coefficient. CKD: chronic kidney disease. COPD: chronic obstructive pulmonary disease. cSAH: convexity subarachnoid hemorrhage. DSA: digital subtraction angiography. DWI: diffusion weighted imaging. ESRD: end-stage renal disease. F: female. GBS: Guillain–Barre syndrome. GTC: generalized tonic clonic. HD: hemodialysis. HIV: human immunodeficiency virus. HTN: hypertension. IVIG: intravenous immunoglobulin. M: male. SBP: systolic blood pressure. MRA: magnetic resonance angiography. RRT: renal-replacement therapy. SAH: subarachnoid hemorrhage. SLE: systemic lupus erythematosus. SWI: susceptibility weighted imaging. T-ALL: T-cell acute lymphoblastic leukemia.

**Table 3:** Joanna Briggs Institute critical appraisal and risk of bias results for case reports/series.

Reference	Q1	Q2	Q3	Q4	Q5	Q6	Q7	Q8	Overall	Risk
Abe et al. (2014) [4]	Y	Y	Y	Y	Y	Y	Y	Y	8	Low
Abraham et al. (2020) [5]	Y	Y	Y	Y	Y	Y	Y	Y	8	Low
Abusabha et al. (2017) [6]	Y	Y	Y	Y	Y	Y	Y	Y	8	Low
Akhondian et al. (2002) [8]	Y	Y	Y	Y	Y	Y	Y	Y	8	Low
Andour et al. (2023) [9]	Y	Y	Y	Y	Y	Y	Y	Y	8	Low
Arai et al. (1997) [10]	Y	Y	Y	Y	Y	N	N	N	5	Moderate
Aridon et al. (2011) [11]	Y	Y	Y	Y	Y	Y	Y	Y	8	Low
Bag et al. (2010) [14]	Y	Y	Y	Y	Y	Y	Y	Y	8	Low
Bălaşa et al. (2015) [15]	Y	Y	Y	Y	Y	Y	Y	Y	8	Low
Ball et al. (2023) [16]	Y	Y	Y	Y	Y	Y	Y	Y	8	Low
Bandeo et al. (2018) [17]	Y	Y	Y	Y	Y	Y	Y	Y	8	Low
Barnaure et al. (2014) [19]	Y	Y	Y	Y	Y	Y	Y	Y	8	Low
Bing et al. (2009) [20]	Y	Y	Y	Y	Y	Y	Y	Y	8	Low
Braatz et al. (2014) [21]	Y	Y	Y	Y	Y	Y	N	N	6	Moderate
Chakroun-Walha et al. (2018) [22]	Y	Y	Y	Y	Y	Y	Y	Y	8	Low
Chaudhari et al. (2014) [23]	Y	Y	Y	Y	Y	Y	Y	Y	8	Low
Chiang et al. (2019) [25]	Y	Y	Y	Y	Y	Y	Y	Y	8	Low
Chou et al. (2004) [26]	Y	Y	Y	Y	Y	Y	Y	Y	8	Low
Decker et al. (2009) [27]	Y	Y	Y	Y	Y	Y	Y	Y	8	Low
Deguchi et al. (2012) [28]	Y	Y	Y	Y	Y	Y	Y	Y	8	Low
Dhawan et al. (2010) [29]	Y	Y	Y	Y	Y	N	N	N	5	Low
Di Stefano et al. (2019) [30]	Y	Y	Y	Y	Y	N	N	N	5	Moderate
Doi et al. [101]	Y	Y	Y	Y	N	N	N	N	4	High
Doi et al. [31]	Y	Y	Y	Y	Y	Y	Y	Y	8	Low
Fujii et al. [32]	Y	Y	Y	Y	Y	Y	Y	Y	8	Low
Gowan et al. [33]	Y	Y	Y	Y	Y	Y	Y	Y	8	Low
Grossbach et al. [34]	Y	Y	Y	Y	Y	Y	Y	Y	8	Low
Hama et al. [35]	Y	Y	Y	Y	Y	Y	Y	Y	8	Low
Han et al. [36]	Y	Y	Y	Y	Y	N	Y	Y	7	Low
Hayashi et al. [37]	Y	Y	Y	N	Y	N	N	Y	5	Mod
Hebant et al. [38]	Y	Y	Y	Y	Y	Y	Y	Y	8	Low
Ho et al. [12]	Y	Y	Y	Y	Y	Y	Y	Y	8	Low
Honda et al. [13]	Y	Y	Y	Y	Y	Y	Y	Y	8	Low
Jesrani et al. [39]	Y	Y	Y	Y	Y	Y	Y	Y	8	Low
Jia et al. [40]	Y	Y	Y	Y	Y	Y	Y	Y	8	Low
Kachi et al. [41]	Y	Y	Y	Y	Y	Y	Y	Y	8	Low
Kitaguchi et al. [43]	Y	Y	Y	Y	Y	Y	Y	Y	8	Low
Lamotte et al. [44]	Y	Y	Y	Y	Y	Y	Y	Y	8	Low
Lee et al. [45]	Y	Y	Y	Y	Y	Y	Y	Y	8	Low
Liu et al. [46]	Y	Y	Y	Y	Y	Y	Y	Y	8	Low
Maciel et al. [47]	Y	Y	Y	Y	Y	Y	Y	Y	8	Low
Maier et al. [50]	Y	Y	Y	Y	Y	Y	Y	Y	8	Low
Malhotra et al. [48]	Y	Y	Y	Y	Y	Y	Y	Y	8	Low
Maruyama et al. [50]	Y	Y	Y	Y	Y	Y	Y	Y	8	Low
Matsumoto et al. [51]	Y	Y	Y	Y	Y	Y	N	N	6	Moderate
McCarron et al. [52]	Y	Y	Y	Y	Y	Y	Y	Y	8	Low
Moosa et al. [53]	Y	Y	Y	Y	Y	Y	Y	Y	8	Low
Nagato et al. [54]	Y	Y	Y	Y	Y	Y	Y	Y	8	Low
Nanba et al. [55]	Y	Y	Y	Y	Y	Y	Y	Y	8	Low
Navarro-Ballester et al. [56]	Y	Y	Y	Y	Y	Y	Y	Y	8	Low
Ocek et al. [57]	Y	Y	Y	Y	Y	Y	Y	Y	8	Low
Ogaki et al. [66]	Y	Y	Y	Y	Y	Y	Y	Y	8	Low
Ohashi et al. [58]	Y	Y	Y	Y	Y	Y	Y	Y	8	Low
Onomura et al. [59]	Y	Y	Y	Y	Y	Y	Y	Y	8	Low
Osman et al. [60]	Y	Y	Y	Y	Y	Y	Y	Y	8	Low
Ou et al. [61]	Y	Y	Y	Y	Y	Y	Y	Y	8	Low
Raya et al. [62]	Y	Y	Y	Y	Y	Y	Y	Y	8	Low
Resorlu et al. [63]	Y	Y	Y	Y	N	N	N	N	4	High
Ribeiro et al. [65]	Y	Y	Y	Y	Y	Y	Y	Y	8	Low
Sallah et al. [64]	Y	Y	Y	Y	Y	Y	Y	Y	8	Low
Sharma et al. [67]	Y	Y	Y	Y	Y	Y	Y	Y	8	Low
Shimizu et al. [68]	Y	Y	Y	Y	Y	Y	Y	Y	8	Low
Srinivasan et al. [69]	Y	Y	Y	Y	Y	Y	Y	Y	8	Low
Tan et al. [70]	Y	Y	Y	Y	Y	Y	Y	Y	8	Low
Tari Capone et al. [71]	Y	Y	Y	Y	Y	Y	Y	Y	8	Low
Thambisetty et al. [72]	Y	Y	Y	Y	Y	Y	Y	Y	8	Low
Tortora et al. [73]	Y	Y	Y	Y	Y	Y	Y	Y	8	Low
Vaysman et al. [75]	Y	Y	Y	Y	Y	Y	Y	Y	8	Low
Wakely et al. [76]	Y	Y	Y	Y	Y	Y	Y	Y	8	Low
Wittgrove et al. [77]	Y	Y	Y	Y	Y	Y	Y	Y	8	Low
Yamagami et al. [78]	Y	Y	Y	Y	Y	Y	Y	Y	8	Low
Yokoyama et al. [79]	Y	Y	Y	Y	Y	Y	Y	Y	8	Low
Zhang et al. [80]	Y	Y	Y	Y	Y	Y	Y	Y	8	Low
Havenon et al. [81]	Y	Y	Y	Y	Y	Y	Y	Y	8	Low
Milia et al. [82]	Y	Y	Y	Y	Y	Y	Y	Y	8	Low
Samara et al. [83]	Y	Y	Y	Y	Y	Y	Y	Y	8	Low
Liu et al. [84]	Y	Y	Y	Y	Y	Y	Y	Y	8	Low
Chan et al. [87]	Y	Y	Y	Y	Y	Y	Y	Y	8	Low
Gocmen et al. [85]	Y	Y	Y	Y	Y	Y	Y	Y	8	Low
Marrone et al. [86]	Y	Y	Y	Y	Y	Y	Y	Y	8	Low
Agarwal et al. [88]	Y	Y	Y	Y	Y	Y	Y	Y	8	Low
Choh et al. [89]	Y	Y	Y	Y	Y	Y	Y	Y	8	Low
Khokhar et al. [90]	Y	Y	Y	Y	Y	Y	Y	Y	8	Low
Srichawla et al. [95]	Y	Y	Y	Y	Y	Y	Y	Y	8	Low

Q1. Were the patient’s demographic characteristics clearly described? Q2. Was the patient’s history clearly described and presented as a timeline? Q3. Was the current clinical condition of the patient on presentation clearly described? Q4. Were diagnostic tests or assessment methods and the results clearly described? Q5. Was the intervention(s) or treatment procedure(s) clearly described? Q6. Was the post-intervention clinical condition clearly described? Q7. Were adverse events (harms) or unanticipated events identified and described? Q8. Does the case report provide takeaway lessons? Overall: Sum of points. Y—Yes; N—No; U—Unclear; NA—Not applicable.

**Table 4:** Assessment of retrospective cohort studies using the Newcastle–Ottawa scale.

Study	Selection (4 Score)	Comparability (2 Score)	Outcome (3 Score)	Total
Ahn et al. (2004) [7]	***	**	***	8
Bansal et al. (2020) [18]	***		*	4
Brewer et al. [97]	***	**	***	8
Chen et al. (2017) [91]	****	**	***	9
Chou et al. (2004) [26]	***	**	***	8
Fitzgerald et al. (2015) [92]	***	**	***	8
Li et al. [98]	***	**	***	8
McKinney et al. (2013) [2]	***	**	***	8
McKinney et al. (2007) [93]	***	**	***	8
Raman et al. (2017) [99]	***	**	***	8
Yoon et al. (2013) [100]	***	**	***	8
Aygunes et al. (2024) [94]	***	**	***	8

### Limitations and Future Direction

This review has several limitations. First, the included studies predominantly consisted of case reports and small retrospective cohort studies, limiting the generalizability of our findings. The heterogeneity observed (I^2^ = 71%, τ^2^ = 0.2046) suggests variability in study designs, diagnostic criteria, and patient populations. Our meta-analysis attempts to address this through random-effects modeling. Additionally, publication bias may have influenced our results, as suggested by the asymmetry in the funnel plot and influential studies identified in the Baujat plot. The reliance on English-language studies may also introduce selection bias. Although we show that cvPRES has a higher mean MAP compared to isolated PRES seen in other studies, head-to-head studies needs to be completed to validate this. Further research is needed to better elucidate the pathophysiology and optimal management of central-variant PRES. Prospective studies with larger sample sizes and standardized diagnostic criteria are essential to validate our findings and explore the impact of specific interventions on patient outcomes. Symptoms and clinical characteristics of cvPRES appear to be similar to those of normal PRES; however, this should be studied using a comparator group. Moreover, longitudinal studies examining the long-term cognitive and functional sequelae of central-variant PRES could provide valuable insights into its prognosis. Finally, the integration of advanced hemodynamic monitoring, particularly those techniques that can measure the rate of MAP changes to estimate cerebral perfusion pressure and autoregulatory changes within the cerebral vasculature, will be necessary to identify the exact pathophysiological underpinnings of cvPRES compared to its typical clinico-radiographic presentation.

## 5. Conclusions

Central-variant PRES (cvPRES) manifests predominantly in younger adults with severe hypertension, most often involving the pons and other central structures (i.e., subcortical nuclei, brainstem, cerebellum, and spinal cord), and has a pooled incidence rate of 13% amongst cohort studies of PRES. Despite higher blood pressures and critical brainstem edema, outcomes are generally favorable (2% mortality), reflecting reversible vasogenic injury rather than permanent neuronal damage. Cases of cvPRES have a higher MAP compared to that what is reported in the literature of typical PRES. However, this needs to be supported with original studies. Early recognition of its distinctive imaging pattern and prompt blood-pressure management are key to optimizing recovery. Future prospective studies should standardize diagnostic criteria, elucidate underlying autoregulatory mechanisms, and evaluate targeted interventions to further improve patient outcomes.

## Figures and Tables

**Table 1:** Databases queried including the search string utilized.

Database	Search String
PubMed/PubMedCentral/MEDLINE	(“Posterior Leukoencephalopathy Syndrome” OR “Posterior Reversible Encephalopathy Syndrome” OR “PRES”) AND (“central variant” OR “central variant” OR “brainstem” OR “spinal cord”)
ScienceDirect	(“Posterior Reversible Encephalopathy Syndrome” OR “PRES”) AND (“central variant” OR “brainstem” OR “spinal cord”)
Scopus	(“Posterior Leukoencephalopathy Syndrome” OR “Posterior Reversible Encephalopathy Syndrome” OR “PRES”) AND (“central-variant” OR “central variant” OR “brainstem” OR “spinal cord”)
Hinari	(“Posterior Leukoencephalopathy Syndrome” OR “Posterior Reversible Encephalopathy Syndrome” OR “PRES”) AND (“central variant” OR “central variant” OR “brainstem” OR “spinal cord”)

## Data Availability

All data generated or analyzed during this study are included in this article. Further enquiries can be directed to the corresponding author.
